# N_2_O emission factors for cattle urine: effect of patch characteristics and environmental drivers

**DOI:** 10.1007/s10705-023-10290-0

**Published:** 2023-07-10

**Authors:** Lena Barczyk, Kate Kuntu-Blankson, Pierluigi Calanca, Johan Six, Christof Ammann

**Affiliations:** 1 Climate and Agriculture Group, Agroscope Research Station, Reckenholzstrasse 191, 8046 Zurich, Switzerland; 2 Department of Environmental Systems Science, ETH Zürich, Universitätstrasse 2, 8092 Zurich, Switzerland

**Keywords:** Cattle urine patch, Nitrous oxide, Pasture, Patch characteristics, Environmental driver

## Abstract

Urine patches from grazing cattle are hotspots of nitrous oxide (N_2_O) emissions. The default IPCC emission factor for urine patches (EF_urine_) is 0.77% for wet climates and 0.32% for dry climates. However, literature reports a considerable range of cattle urine EF values and urine characteristics used in experimental studies, revealing contrary results on the effects of urine patch characteristics and seasonal pattern. Therefore, we examined N_2_O emissions and corresponding EF_urine_ values in relation to urine patch characteristics (urine N concentration, urine volume, patch area, urine composition) and environmental drivers (precipitation, water filled pore space, soil temperature). Ten artificial urine application experiments were performed from July 2020 to June 2022 on a pasture located in Eastern Switzerland. Urine N concentration, patch area, volume and urine N composition showed no significant effects on the EF_urine_ value (p > 0.05). EF_urine_ varied, however, strongly over time (0.17–2.05%). A large part of the variation could be predicted either by cumulative precipitation 20 days after urine application using a second order polynomial model (Adj. R^2^ = 0.60) or average WFPS 30 days after urine application using a linear model (Adj. R^2^ = 0.45). The derived precipitation model was used to simulate EF_urine_ weekly over the last 20 years showing no significant differences between the seasons of a year. The resulting overall average EF_urine_ was 0.67%. More field studies are needed across sites/regions differing in climate and soil properties to implement a country-specific EF_3_ for Switzerland and to improve the quantification of N_2_O emissions at the national scales.

## Introduction

There is a broad consensus that anthropogenic-driven climate change is due to excessive greenhouse gas emissions (GHG) and associated changes in atmospheric composition since industrialization. The concentration of nitrous oxide (N_2_O), a powerful GHG with a global warming potential of 298 CO_2_ equivalent and stratospheric ozone depleting molecule, has been increasing steadily in the troposphere (Yu et al. 2020; Park et al. 2012). Agriculture is responsible for 60% of global anthropogenic N_2_O emissions, mainly due to the use of nitrogen (N) fertilizers and productive livestock (Mosier et al. 1998; IPCC 2006; Syakila and Kroetze 2011). In consideration of a growing and ever-more demanding human population, more food and agricultural area will be needed. Consequently, N_2_O emissions are expected to continue rising (Tian et al. 2018).

Aerobic autotrophic nitrification and anaerobic heterotrophic denitrification in soils contribute to approximately 70% of global N_2_O emissions (Butterbach-Bahl et al. 2013; Braker and Conrad 2011; Davidson 1991; Congreves et al. 2019). Studies have shown that in agricultural soils peak N_2_O emissions follow N fertilizations, assuming a linear or even an exponential response of N_2_O emissions to increasing N input (Kim et al. 2010, 2013; Shcherbak et al. 2014). Especially in grazed pasture systems, N input rates locally exceed the potential plant N uptake very easily due to the urine patches of grazing cattle that contain 200 to 2000 kg N ha^−1^ and thus inducing substantial N_2_O emissions (Welten et al. 2013; Selbie et al. 2015; Haynes and Williams 1993).

In the IPCC Tier 1 approach, N_2_O emissions from agricultural soils are described using annual average emission factors (EF) that represent the fraction of N inputs emitted as N_2_O. The global default EF value for grazing based N inputs for cattle EF_3PRP,CPP_ (subsequently named EF_3_) of 2% was recently replaced by a new global value of 0.4% disaggregated into wet (EF_3_: 0.6%) and dry climate regions (EF_3_: 0.2%) (IPCC 2019). Effectively, higher precipitation is linked to higher soil water content known as a driver of N_2_O emissions by regulating the oxygen availability to microbes.

The calculation and reporting of grazing based N_2_O emissions in national GHG inventories by using a global default EF_3_ value involve uncertainties because it is based on limited studies that may not reflect regional conditions. The international project DATAMAN collated a database of N_2_O EF_3_ data revealing an imbalance in regional representativeness with 56%, 18%, 6%, and 6% of the total dataset derived from New Zealand, United Kingdom, Kenya and Brazil, respectively (Beltran et al. 2021). In order to improve the quantification of N_2_O emissions at the national scale, the IPCC advises countries to adopt higher tier methodologies like a country-specific EF_3_ (Tier 2 approach) and to use further disaggregation.

Based on sufficient emission measurements and activity data, Canada and New Zealand were able to disaggregate Tier 2 EF_3_ by province and slope-class (Rochette et al. 2014; van der Weerden et al. 2020). A disaggregation by season has been addressed in a few studies (Krol et al. 2016; Chadwick et al. 2018; Zhu et al. 2021). Seasonal effects could be an important finding for the mitigation of N_2_O emissions, e.g. through seasonal grazing or lower stocking densities (Monaghan and de Klein 2014). Temporal variations of N_2_O emissions are often linked to changing meteorological conditions (Rowlings et al. 2015). Krol et al. (2016), for instance, associated high EF_3_ values with high rainfall and high soil moisture conditions during autumn at three field sites of contrasting soils across Ireland. However, the literature does not provide a consistent seasonal pattern and a seasonal disaggregation of EF_3_ is difficult due to inconsistent seasonal effects in temperate climates compared to tropical regions, which exhibit more clear wet versus dry seasons (Mancia et al. 2022).

Generally, the N_2_O EF for cattle urine patches (subsequently named EF_urine_) is significantly higher than for dung (subsequently named EF_dung_) (Krol et al. 2016; Chadwick et al. 2018) and thus of main importance. Therefore, IPCC (2019) recommends the use of individual EF values, proposing a default EF_urine_ of 0.77% for wet climates and 0.32% for dry climates. A meta-analysis revealed a range of EF_urine_ values of 0.01% to 5.50% (López-Aizpún et al. 2020), which exceeds the uncertainty range of the default EF_urine_ values and highlights the need to use higher Tiers (IPCC 2019). Only few countries like Ireland, Japan or the UK implemented a disaggregation of the EF_3_ based on the animal excreta type.

Cattle urine N concentration can vary strongly from 1 to 20 g N L^−1^ (Oenema et al. 1997) and is positively correlated with the feed N intake. The cattle N use is generally inefficient as 70–95% of ingested N is excreted (Oenema et al. 2005) but it can be improved by feeding supplements with lower crude protein and higher carbohydrate content (Firkins and Reynolds 2005; Talbot et al. 2020; Dalley et al. 2017). Van Vuuren and Smits (1997) found an increase in urination volume by changing the diet from a low to high N intake while the urine N concentration stayed constant. In addition, the amount of urine production is positively affected by the intake of Na and K (De Campeneere et al. 2006; Bannink et al. 1999). Cattle urine includes a variety of nitrogenous constituents among which urea accounts for the largest fraction and it generally increases with increased dietary protein intake.

To investigate seasonality and environmental effects on EF_urine_ (and/or EF_3_) values, the use of standardized urine patches is advantageous, because N_2_O emissions are also influenced by urine patch characteristics. Differing urine patch characteristics have been used in studies, Haynes and Williams (1993) for instance proposed a typical urine deposit of 2.0 L onto an area of 0.2 m^2^ containing 20 g N. However, rates of urine N exceed the possible plant N intake, hence soil N surplus might be used for N_2_O production and might result in a nonlinear exponential response of N_2_O emissions to the N input (Kim et al. 2013). Furthermore, higher urine volumes may favour anaerobicity in the topsoil and, thus, inhibit nitrification while increasing the N_2_/N_2_O ratio (van Groeningen et al. 2005a) or lead to a deeper infiltration of urine, thus lowering the fraction of N remaining in the topsoil (Sordi et al. 2014).

Only a limited number of studies have been carried out observing the effect of the urine volume and the amount of urine N on N_2_O EF_urine_ and values exhibit non-conclusive or even contradictory results. De Klein et al. (2014) showed an increasing trend of EF_urine_ by increasing the urine N concentration (in equal urine volume) while Selbie et al. (2014) observed a decreasing EF_urine_. Da Silva Cardoso et al. (2016) found a linearly decreasing EF_urine_ by increasing the urine volume (with same N concentration) but there was no effect observed in Pal Singh et al. (2021). Also, van Groeningen et al. (2005b) saw a significant effect of different urine volumes containing a similar amount of urine N only in single application experiments. Besides real cattle urine, a variety of synthetic urines containing differing fractions of N constituents have been used (Petersen et al. 2005; de Klein et al. 2003; Kool et al. 2006b). The effect of nitrogenous urine composition on N_2_O emission has been addressed in few studies; Kool et al. (2006a), for instance, showed that increased hippuric acid contents in urine lowered N_2_O emissions.

A very limited number of measurement campaigns have been carried out in Central Europe to derive EF_urine_ values, hence countries need to use IPCC default EF values for the quantification of urine derived N_2_O emissions. In this study, we conducted N_2_O measurements at a Swiss pasture site. To our knowledge, this is the first study of this extent conducting repeated measurements over the entire grazing season to investigate seasonal and environmental effects on the EF_urine_ value. Literature shows non-conclusive or contrary results in the effects of urine patch characteristics. Therefore, we also assessed the effect of various urine patch characteristics, namely whether the EF_urine_ is affected by (1) urine N concentration when keeping the volume constant, (2) the patch area, (3) urine volume when keeping the urine N concentration or the total urine N input constant, and (4) urine N composition.

## Methodology

### Study site

The present study was conducted from July 2020 to June 2022 in a dairy pasture system near the research station Agroscope Tänikon (47°29′26.7″N, 8°55′12.1″E; 517 m elevation) located in North-Eastern Switzerland. The climate is temperate with a long-term (2009–2019) average annual air temperature of 9.5 °C and an annual precipitation amount of 1124 mm (derived from the Federal Office of Meteorology and Climatology “MeteoSwiss”, weather station Tänikon) (MeteoSwiss 2022). The field side has been a permanent pasture system since 2013. In 2015 it was sown with a standard mixture for feed production (UFA 440) consisting of *Trifolium repens, Trifolium pratense, Festuca rubra* agg., *Lolium perenne, Poa pratensis* and *Phleum pratense*. In addition, it was over-sown with *Lolium perenne* in 2016 and 2019. The soil is classified as Luvisol (FAO classification system, IUSS 2022) with a loamy texture. The topsoil contained 14% clay, 42% silt, 39% sand, 5.2% organic matter, 2.46% carbon and 0.24% nitrogen. The soil had a pH of 6.3, bulk density of 1280 kg m^−3^ and a pore volume of 51% (10 sample replicates from 50 to 100 mm depth). The pasture of 2.8 ha was rotationally grazed from April to October by Brown Swiss and Red Holstein dairy cattle. Each year, a different area of roughly 0.13 ha for the urine application experiments was fenced off and excluded from grazing to minimize the influence of old excreta patches. Thus, the start of each application experiment was ≥ 6 months after the last grazing activity of the previous year.

### Experimental design

Within the fenced off area of the pasture, cattle urine patches were simulated by controlled application of synthetic and real urine in ten experiments within the 2-year study period. A randomised design was used with a minimum of 3 repetitions per treatment and a distance between individual patches of about 1.5 m. For experiments with only few different treatments the number of repetitions was increased up to 6. We predominantly used synthetic urine for having a better control over the exact composition. It was produced one day before application and stored overnight at 4 °C. The standard synthetic urine contained urea (91% of N) and hippuric acid (9% of N), as well as 14 g KHCO_3_, 10.5 g KCl, 0.4 g CaCl_2_*2H_2_O, 1.2 g MgCl*5H_2_O and 3.7 g Na_2_SO_4_ [L^−1^] to mimic real urine properties as suggested by Kool et al. (2006b). At three occasions, also real urine was applied that had been collected from the dairy cattle in the nearby barn one hour before. Table 1 gives an overview of conducted experiments U1 to U10. In all experiments, two litres of standard synthetic urine containing 20 g of N was uniformly applied to a circular area of 0.12 m^2^ corresponding to a rate of 1667 kg N ha^−1^ and 17 mm water addition. In addition, varying urine volumes of two to three levels were applied in U2, U3 and U5, while in U7 and U10 differing urine N concentrations were compared. To examine the effect of the liquid addition via urine, differing urine volumes with the same total N were compared in U10. In U8, the standard synthetic urine was applied to three differing areas of 0.12, 0.25 and 0.36 m^2^. Comparative to the standard synthetic urine composition, a simpler urine solution containing only urea (100% of N) as N compound and a more complex urine solution containing urea (76.7% of N), hippuric acid (5.1% of N), allantoin (14.1% of N), uric acid (0.8%) and creatinine (3.2%) were produced according to Kool et al. (2006b) in U9. Grass was cut one to two weeks before the experiment starts and every 4–6 weeks afterwards depending on growth conditions.

**Table 1:** List of conducted experiments (U1-U10). The synthetic “standard” urine was applied in 2 Liters of 20 gN to an area of 0.12 m^2^ with urea and hippuric acid as N constituents. Various urine patch characteristics were assessed by comparing varying (1) urine N concentrations in U1 and U10, (2) urine patch areas in U8, (3) urine volumes having the same N concentration or the same total N in U2, U3, U5 and U10, and (4) synthetic urine N compositions in U9

Experiment	Application date	Measurement length (days)	Treatment	Urine volume (L)	Nitrogen applied (g patch^−1^)	Patch area (m^2^)	Urine composition	Nitrogen application rate (kg N ha^−1^)	Urine pH-value
All experiments			Control		0			0	
U1	09.07.2020	125	Standard	2	20.0	0.12	Standard	1667	7.0
U2	20.08.2020	80	Standard	2	20.0	0.12	Standard	1667	7.0
		80	Real 2 L	2	11.4	0.12	Real urine	946	8.4
		80	Real 1 L	1	5.7	0.12	Real urine	473	8.4
U3	20.10.2020	26	Standard	2	20.0	0.12	Standard	1667	7.0
		26	1 L	1	10.0	0.12	Standard	833	7.0
U4	06.05.2021	89	Standard	2	20.0	0.12	Standard	1667	7.0
		89	Water only			0.12			7.0
U5	10.06.2021	128	Standard	2	20.0	0.12	Standard	1667	7.0
		128	Real 1 L	1	4.6	0.12	Real urine	381	8.4
		128	Real 2 L	2	9.1	0.12	Real urine	762	8.4
		128	Real 3 L	3	13.7	0.12	Real urine	1143	8.4
U6	06.08.2021	71	Standard	2	20.0	0.12	Standard	1667	7.0
U7	19.08.2021	58	10 gN	2	10.0	0.12	Standard	833	7.0
		58	Standard	2	20.0	0.12	Standard	1667	7.0
		58	30 gN	2	30.0	0.12	Standard	2500	7.0
U8	08.09.2021	48	Standard	2	20.0	0.12	Standard	1667	7.0
		48	0.25 m^2^	2	20.0	0.25	Standard	800	7.0
		48	0.36 m^2^	2	20.0	0.36	Standard	556	7.0
U9	22.09.2021	42	Standard	2	20.0	0.12	Standard	1667	7.0
		42	Urea only	2	20.0	0.12	Urea only	1667	8.1
		42	Complex	2	20.0	0.12	Complex	1667	7.1
		42	Real	2	16.7	0.12	Real urine	1392	8.3
U10	14.05.2022	42	4 gN	2	4.0	0.12	Standard	333	7.0
		42	Standard	2	20.0	0.12	Standard	1667	7.0
		42	2 L,10 gN	2	10.0	0.12	Standard	833	7.0
		42	3 L,10 gN	3	10.0	0.12	Standard	833	7.0
		42	1 L,10 gN	1	10.0	0.12	Standard	833	7.0

### Chamber measurements

N_2_O emissions were measured by one large opaque manual chamber of 0.8 m × 0.8 m × 0.5 m named fast-box (FB) according to Voglmeier et al. (2019). It covered the wetted patch area and some surrounding area. The ecosystem was disturbed in a minimized way as no frame was inserted into soil. Instead, the FB base was sealed against the pasture surface by a foam band of 50 mm compression width that can easily adapt to the unevenness of the surface. A vent tube of 25 mm diameter and 200 mm length filled with foam was installed at the FB for air pressure equilibration but avoiding uncontrolled air exchange due to wind-induced pressure fluctuations. Additionally, the FB was equipped with a GMP343 CO_2_ probe (Vaisala, FI) for quality control of the measurements. Air from the headspace of the FB chamber was continuously sampled through a 30 m polyamide tube to a fast response quantum cascade analyser (QCL, Aerodyne Research Inc.) measuring N_2_O concentrations. The sample flow rate was around 9.5 L min^−1^. The increase of gas concentrations in the chamber headspace was recorded every three seconds for the total FB closure time of 120–130 s. FB measurements were conducted one to two hours past urine application, then daily to once every two days in the first week, followed by a weekly and once every two weeks measuring interval until N_2_O fluxes of urine patches had gone back to the level of the control measurements on untreated areas. For applications in the early grazing seasons, fluxes could be measured longer (until the end of the grazing season) while experiment length was shorter for applications in the late grazing season (autumn). To reflect the daily average N_2_O fluxes, measurements were carried out between 10:00 am and 14:00 pm (Charteris et al. 2020).

During chamber closure and for every individual patch, volumetric soil moisture content (VWC) of the soil surface soil layer was measured with a GS3 probe (Decagon Devices Inc.) inserted vertically from the surface (0–55 mm depth). Furthermore, VWC and soil temperature profiles were measured continuously at the experimental site using GS3 (Decagon Devices Inc.) and ML3 (Delta-T Devices Ltd.) probes, from which the 50 mm level was used for the present study. Based on VWC, we derived water-filled pore space (WFPS) using total pore volume determined from soil samples taken at the site. In addition, a weather station installed in the experimental area recorded typical meteorological variables including precipitation.

### Flux calculation and quality control

Owing to its design, the manual FB is supposed to experience a minor inflow of surrounding background concentration C_bg_ air slightly lowering the increase of gas concentrations in the chamber headspace (Voglmeier et al. 2019). To account for this effect, the measured gas concentration C_meas_(t) in the chamber headspace during closure was corrected by the following formula for the subsequent flux calculation

1 $$C\left( t \right) = C_{meas} \left( t \right) + \left[ {\left( {C_{meas} \left( t \right) - C_{bg} } \right)\cdot \frac{Q}{V}t} \right]$$

with the corrected gas concentration C, chamber volume V, sample flow rate Q and the time since closure t. FB gas fluxes were calculated with the HMR package in R (Pedersen et al. 2010) using linear and nonlinear regression. According to Pedersen et al. (2010), unknown effects by chamber leaks or by the disturbance of the concentration gradient in the soil lead to a deviation from the linear increase in the chamber headspace concentration that can be described by an exponential saturation function shape with parameter $\varkappa$ (kappa), chamber height h, chamber equilibrium concentration C_eq_ and initial flux F_0_

2 $$C\left( t \right) = C_{eq} + F_{0} \frac{{{\text{exp}}\left( { - \varkappa t} \right)}}{ - \varkappa h}$$

3 $$F_{0} = h\frac{dC}{{dt}}|t = 0$$

The initial flux F_0_ ≡ F_nonlinear_ is supposed to represent the true emission flux without chamber disturbance effects. In addition, for each case also a linear fit for dC/dt over the entire chamber closure time was calculated resulting in a corresponding flux F_linear_. The effective minimal detectable flux (MDF) was determined by a statistical approach using all positive fluxes that were not significantly different from zero based on their 95% confidence intervals (CI). 95% of flux subsample data points fell below 15 μg N_2_O-N m^−2^ h^−1^ thus assigned as MDF. Fluxes below the MDF were always calculated linearly and fluxes above the MDF were calculated using the automated model selection of the HMR package. Respective fluxes were rejected if quotations of F_nonlinear_/F_linear_ > 4 (Hüppi et al. 2018) or if the coefficient of determination (R^2^) of the corresponding CO_2_ flux was < 0.8 (CO_2_ -criterion). In addition, N_2_O fluxes were rejected if the relative uncertainty was ≥ 25% and the absolute uncertainty > MDF. Overall, 28% of the fluxes were calculated by nonlinear regression, 65% by linear regression and 7% of calculated fluxes were not considered for further analysis.

The common chamber flux calculation formula (Eq. 3) yields a trace gas flux per unit surface area (e.g. per m^−2^ or ha) implicitly assuming a spatially uniform flux. Multiplication with the chamber surface area (0.64 m^2^) yields the total emission flux under the chamber. This is a more meaningful quantity for the present study, because our measurement chamber covered an area larger than the urine patches. Therefore, the flux under the chamber is not spatially uniform but comprises the total urine patch emission, including potential edge effects, and the background flux of the area covered by the chamber. The background flux was determined separately for each experiment by control measurements on untreated areas (without urine patch). Since the individual urine patch is the relevant unit and strongly dominating emission source in this study (one to two orders of magnitude larger than the background flux), we generally present the emission measured by chamber in units of g N_2_O-N patch^−1^.

### Data analysis and statistics

Data analysis was performed using the R software (version 3.1.3). When having more than one flux per treatment replicate and day, the best flux according to the CO_2_ -criterion was used. FB fluxes obtained were assumed to be representative on average for the day of measurement and were converted to daily fluxes. For estimating cumulative N_2_O emissions, daily fluxes were linearly interpolated for every treatment replicate separately. Cumulative fluxes were averaged per treatment. The uncertainty of these average results were dominated by the (spatial) variability among the repetitions indicated either as standard error or 95% CI. Also, N_2_O emission factors for urine patches $\text{EF}_{\text{urine}}$ were calculated for every treatment replicate separately as the difference between the average cumulative treatment flux ${\text{N}}_{2} {\text{O}}_{\text{Treatment}}$ and the control flux ${\text{N}}_{2} {\text{O}}_{\text{Control}}$ (in units of g N_2_O-N patch^−1^) divided by the amount of N contained in applied urine $\text{N}_{\text{applied}}$ (in units of g N patch^−1^):

4 $$EF_{urine} = \left( {\frac{{ N_{2} O_{ Treatment} - N_{2} O_{Control} }}{{N_{applied} }}} \right)$$

A one-way ANOVA was performed for testing significant difference of EF_urine_ values among urine treatment levels within each experiment and of seasonally averaged simulated EF_urine_ values. Furthermore, a Welch t-test (for unequal variances) was used to test whether cumulative N_2_O fluxes differ significantly from the corresponding control fluxes (significance threshold at p = 0.05).

To analyse the relationship between EF_urine_ values or N_2_O emissions and possible drivers (average soil WFPS, average soil temperature, cumulative precipitation) a generalized additive model (GAM) function in R (MGCV package) was used. Linear and non-linear models were fitted for single drivers, varying lengths of observation and various combinations without specifying a particular shape a-priori. The output of the non-parametric curve fitting gave the dimension of basis as well as p-values and coefficient of determination. The parameter combinations with the best performance was selected for a linear or polynomial regression.

## Results

### Cumulative N_2_O emissions, EF_urine_ values and environmental variables

**Fig. 1:**
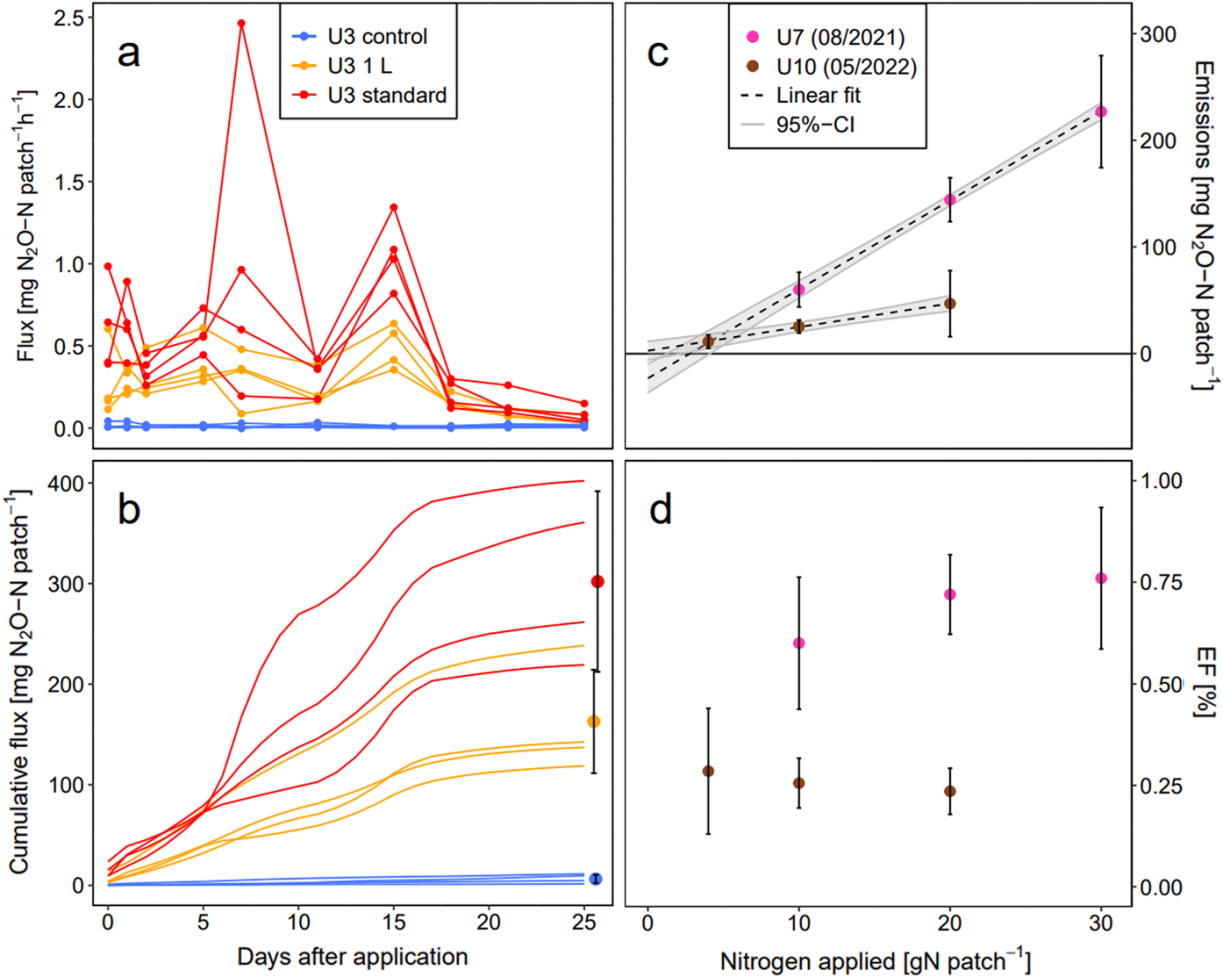
Exemplary time series (U3) of **a** measured N_2_O flux with linear interpolation between data points and **b** the resulting cumulative emission curves for each treatment replicate and the final average emission sums per treatment (with 95% CI). **c** Average cumulative urine emission (control fluxes subtracted) and **d** average EF_urine_ values (including 95% CI) per treatment level of U7 and U10 with regard to the nitrogen applied

Figure 1a, b exemplarily demonstrates the calculation of cumulative N_2_O emissions and EF_urine_ values of single applications as explained in the Methodology section. All cumulative N_2_O emissions and EF_urine_ values of experiments U1–U10 are listed in Table 2. Cumulative N_2_O emissions of untreated control areas ranged from 3.3 to 47.9 mg N_2_O-N patch^−1^, while cumulative N_2_O emissions of urine patches ranged from 14.7 to 430.8 mg N_2_O-N patch^−1^. The corresponding EF_urine_ values of urine treatments varied between 0.17% and 2.05%.

**Table 2:**
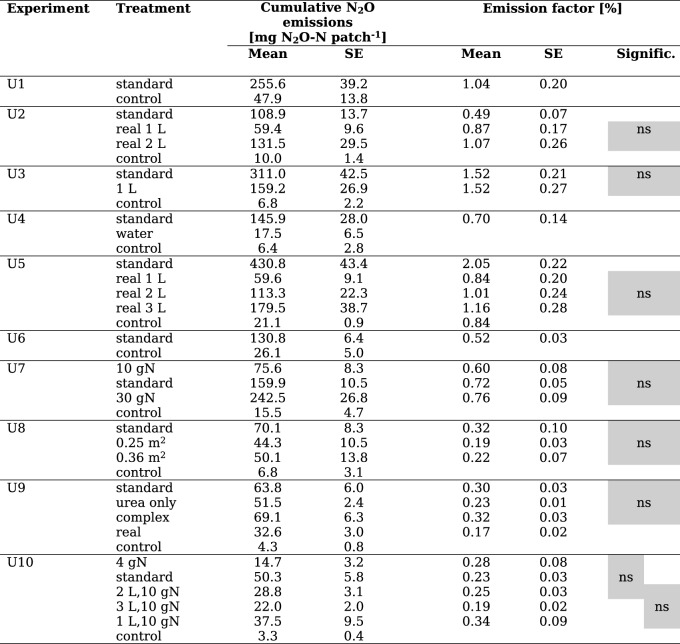
Mean and standard error (SE) of cumulative N_2_O emissions and resulting EF values observed in conducted experiments (U1–U10). Patch-related emissions (including control treatments without patch) refer to the chamber area of 0.64 m^2^. A detailed characterization of the treatments is given in Table 1. Significance of EF results between respective treatment levels is shown (ns: not significant)

In all experiments, cumulative N_2_O emissions of urine treatments were significantly higher (p < 0.05) than cumulative control (non-urine) emissions. Cumulative emissions increased linearly (slope of linear models ≠ 0, p < 0.05) with the urine N concentration (U7, U10), the confidence interval of the intercept enclosing the point of origin (Fig. 1c). The impact of urine N concentrations on EF_urine_ was not significant (Fig. 1d, Table 2). Furthermore, no significant effect of the urine patch area (U8), urine volume (U2, U3, U5), urine liquid addition (U10) and nitrogenous urine composition (U9) on the EF_urine_ was observed (Table 2).

For the differing timings of urine application in U1–U10, precipitation was summed and soil temperature and WFPS were averaged over varying period lengths as explained in the Methodology section. Based on the R^2^ value of the selected model, cumulative precipitation over 20 days past urine application and WFPS averaged over 30 days past urine application showed the best relation with EF_urine_. The soil temperature showed no relation with derived EF_urine_ values.

**Fig. 2:**
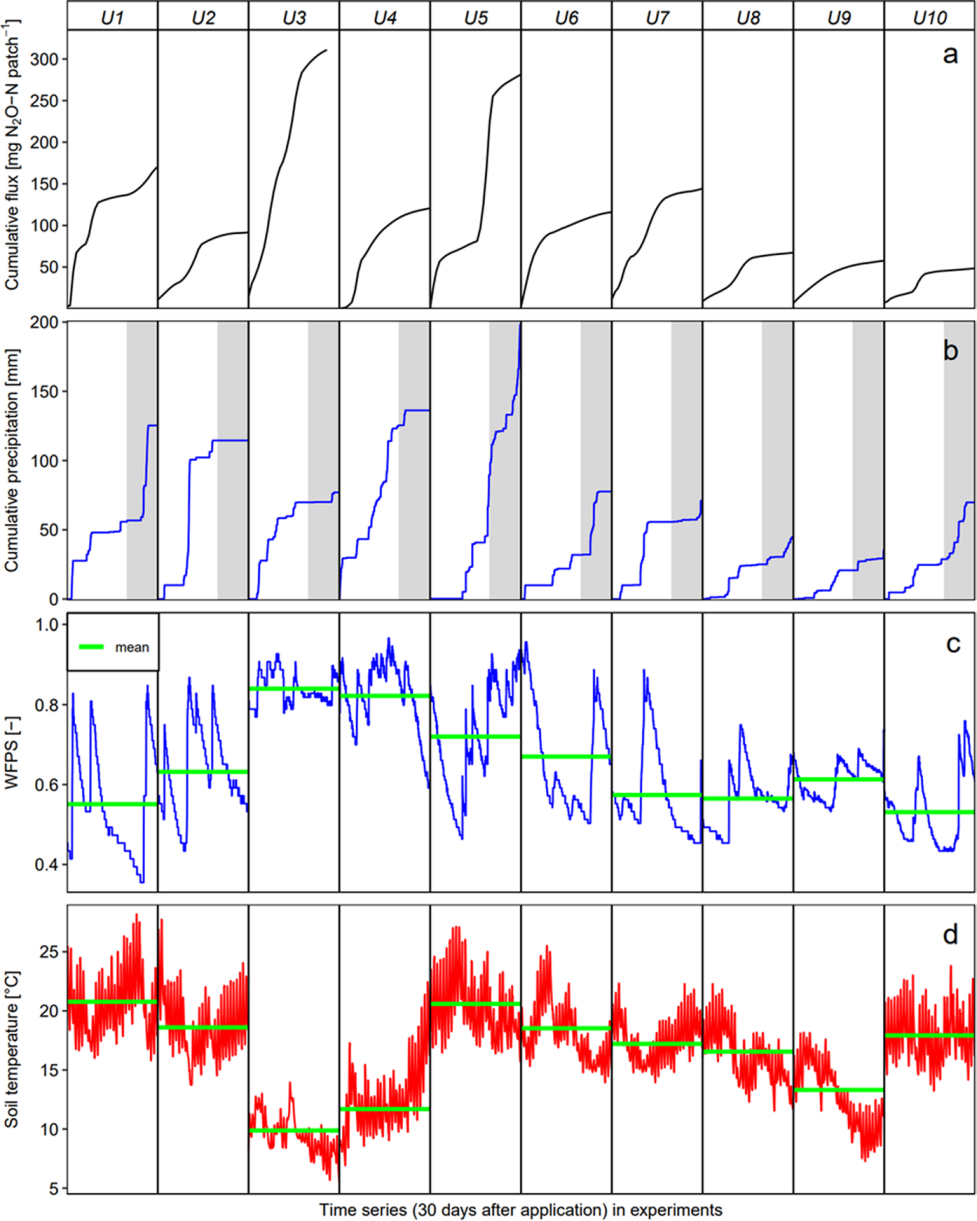
Time series over 30 days past urine application of **a** averaged cumulative N_2_O fluxes of the standard urine, **b** cumulative precipitation, **c** WFPS and **d** soil temperature in 50 mm depth of experiments U1–U10. For statistical modelling of EF_urine_ optimum time periods of 20 days for cumulative precipitation (without grey shading) and 30 days for average soil moisture were used

EF_urine_ values of synthetic standard urine varied from 0.23% to 2.05% within the study period (July 2020 to June 2022). In addition, cumulative precipitation over 20 days past urine application, average soil WFPS 30 days past urine application and average soil temperature 25 days past urine application varied among the experiments (Fig. 2). Experiments U2 and U4 showed the highest cumulative precipitation (> 100 mm), while in U8, U9 and U10 precipitation was lowest (≤ 30 mm). Average WFPS ranged from 0.53 to 0.84, though, in U3 and U9 the WFPS stayed relatively stable (SD ≤ 0.05) whereas in U1, U2, U5, U6 and U7 the WFPS varied more (SD > 0.10). Average soil temperatures ranged from 9.9 °C to 20.8 °C.

### Statistical model results of emission factor and cumulative urine emissions

As urine patch characteristics (urine N concentration, volume, patch area, N composition) had no effect on the EF_urine_, all derived EF_urine_ values were used to parametrise the relationship between EF_urine_ value and environmental variables. Cumulative precipitation over 20 days past urine application and average WFPS over 30 days past urine application showed the best relation with EF_urine_.

**Fig. 3:**
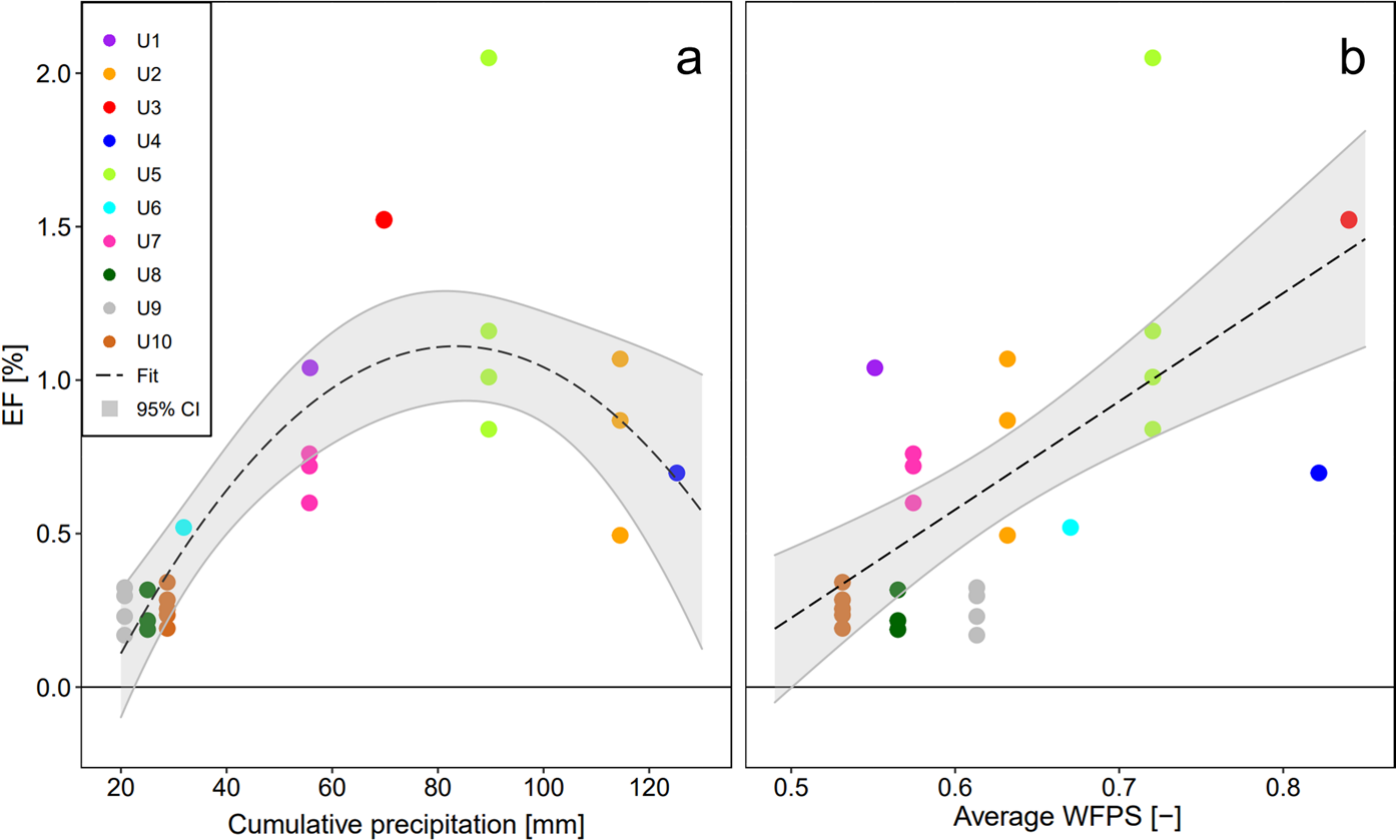
Dependence of observed EF_urine_ values **a** on cumulative precipitation 20 days past urine application and **b** on average WFPS 30 days past urine application. The different time windows for precipitation and WFPS were chosen based on individual optimization (see Methodology section)

The dependence of the EF_urine_ [%] on cumulative precipitation 20 days past urine application P [mm] was described by a second order polynomial regression (Fig. 3), giving highest EF_urine_ values of 1.1% at cumulative precipitation of 83 mm dipping down for lower and higher P:

5 $$EF_{urine} = 0.0415 \cdot P - 0.00025 \cdot P^{2} - 0.621 \quad \left( {{\text{Adj}}.{\text{R}}^{{2}} = 0.{6}0,\;p < 0.000{1}} \right)$$

The relationship between the EFurine (%) and average WFPS [−] over 30 days past urine application was found linear:

6 $$EF_{urine} = 3.53 \cdot WFPS - 1.54\quad \left( {{\text{Adj}}.{\text{R}}^{{2}} = 0.45,\;p < 0.000{1}} \right)$$

To test if soil temperature had an additional effect on the EF_urine_ besides precipitation and WFPS respectively, a correlation analysis (Pearson) was performed between average soil temperature and residuals of the respective model. Correlation coefficients were -0.32 for residuals of the precipitation model (p ≥ 0.05) and 0.46 (p < 0.05) for residuals of the WFPS model. However, both correlations were just moderate (0.3–0.5). We further conducted smoothing spline regression using GAM function in R (package MGCV). Soil temperature was used together with precipitation or WFPS as predictor variables (also in a mixed model and by using differing time periods of predictor averaging/summating), which entailed no improvement on the WFPS and precipitation models.

### Simulation of urine EFs over the grazing season

**Fig. 4:**
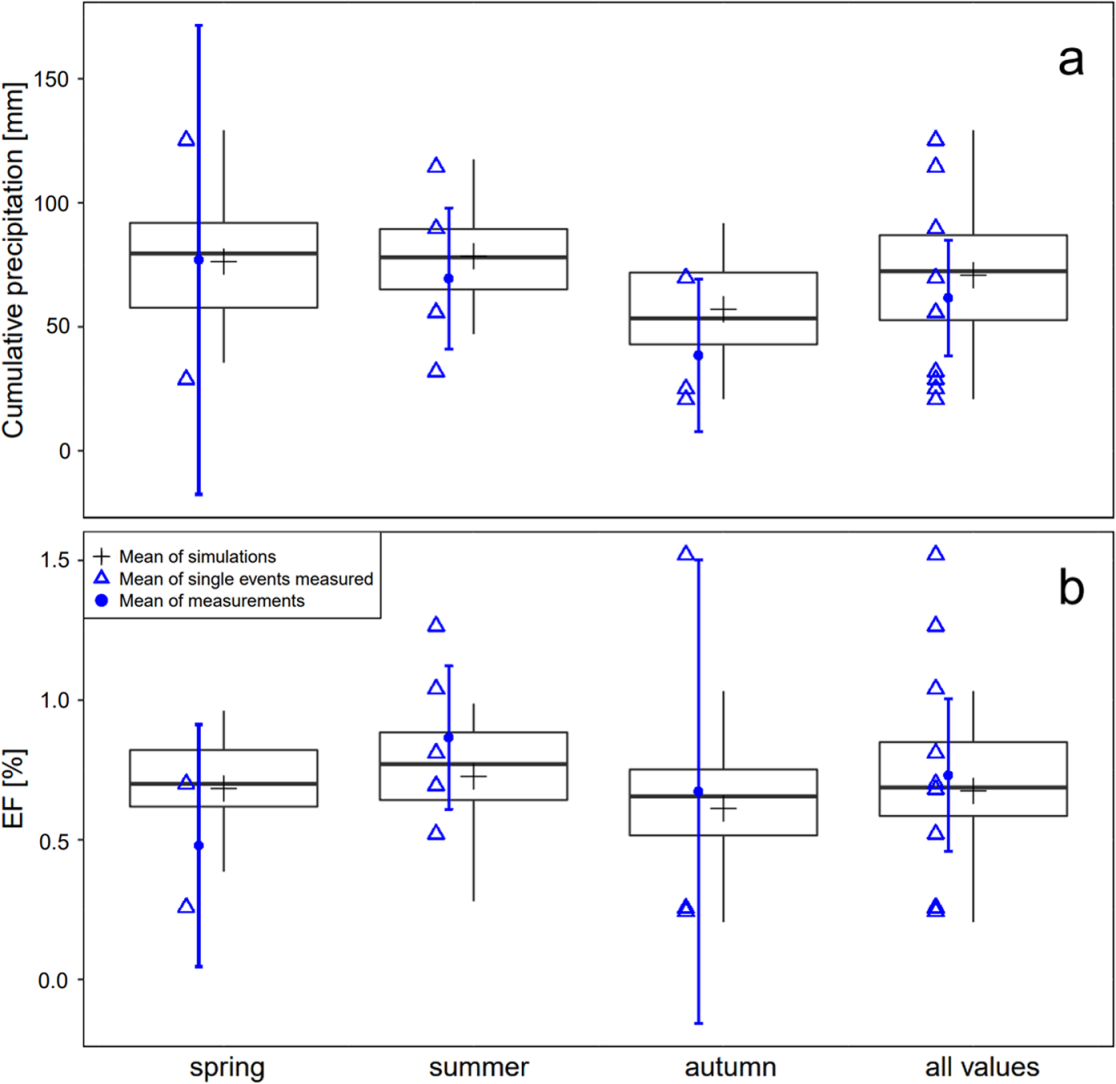
Boxplots of **a** seasonal averaged cumulative precipitation (sum of 20 days) and **b** modelled EF_urine_ values using derived precipitation model (Eq. 5). EF_urine_ was simulated weekly over the entire grazing period (01.04.–31.10.) for the years 1999–2022. Also mean of single experiments and the corresponding average per season including 95% CI is shown

The precipitation model and precipitation data of the MeteoSwiss weather station Tänikon (approx. 1.6 km distance to the study site) were used to simulate EF_urine_ values weekly over the entire grazing period (01.04.–31.10.) for the years 1999–2022 and for disaggregation into seasons (spring: April, May; summer: June, July, August; autumn: September, October). Autumn showed the lowest mean cumulative precipitation over 20 days (57.1 mm), while cumulative precipitation was higher for spring (76.3 mm) and summer (78.5 mm), though with a considerable interannual variability. Seasonally averaged simulated EF_urine_ values were not significantly different ranging from 0.61% ± 0.05 (mean ± standard error of the mean) in autumn to 0.68% ± 0.04 in spring and 0.73% ± 0.04 in summer (Fig. 4). Cumulative precipitation and respective EF_urine_ values of experiments U1–U10 lie in the range of simulated data. The EF_urine_ averaged over the entire period of simulation (0.67% ± 0.05) was slightly lower than the mean of observed EF_urine_ 0.73% ± 0.14.

## Discussion

### N input had no significant effect on EF_urine_

An EF value relates the quantity of N_2_O emissions with the amount of N deposited (IPCC 2006). In this study, urine N loading rates of 333 to 2500 kg N ha^−1^ within patches were applied being in the range of literature reported cattle urine N loading rates (Krol et al. 2016; Selbie et al. 2014; Haynes and Williams 1993). We found no significant differences between EF_urine_ results for varying urine N inputs confirming the assumptions of a constant EF_urine_ (Fig. 1c, d). Therefore, we conclude that the EF_urine_ results obtained from the standard applications (a loading rate of 1677 kg N ha^−1^ is high compared to most other studies in the literature) are valid also for lower N loading rates. However, literature shows conflicting findings for EF_urine_ values to increasing urine N inputs. Van Groeningen et al. (2005a) neither found a significant effect of urine N on EF_urine_ values. Other studies observed that the EF_urine_ value increased by increasing urine N inputs (Singh et al. 2009) or fertilizer N inputs (Cardenas et al. 2010; Zhang and Han 2008) often explained by excessive N supply beyond possible plant uptake that results in soil residual N being used in N_2_O forming microbial processes (Kim et al. 2013). Clough et al. (2003) and Selbie et al. (2014), on the other hand, found a decreasing EF_urine_ with increasing the urine N content. A possible explanation is the limitation of soil microbial N uptake, e.g. through soil C availability (Kim et al. 2013; Selbie et al. 2014). The contradictory findings may be explained by climate conditions and soil properties (e.g. soil aeration, organic and mineral N, availability of C, texture). The explanations given in the literature would imply that in this study N_2_O formation is controlled by competitive urinary N uptake of plants and microorganisms.

### Patch are had no significant effect on EF_urine_

N loading rates of cattle urine patches are higher than in “normal” N fertilizations and exceed the potential plant N uptake (Wheeler et al. 1997). For this reason, we assumed differing EF_urine_ values for different sized urine patches and N loading rates [kg N ha^−1^] respectively, as in a smaller patch the possible N plant uptake is exceeded more easily. The same amount of urine was uniformly applied to areas of 0.12, 0.25 and 0.36 m^2^ covering a wide range of reported urine patch areas (Selbie et al. 2015; Williams and Haynes 1994). However, the effect of the patch size on the EF_urine_ value was not significant in our study, which is similar to findings of Marsden et al. (2016) and van Groeningen et al. (2005a). It has to be considered that the effective patch area is bigger than the wetted area on which the urine was directly applied. The effective area includes the wetted area and the area outside the wetted area in that plants can access urinary N through roots and N diffusion through the soil (Selbie et al. 2015). The concentration gradient between wetted area and untreated soil is expected to be higher in smaller patches (by having the same N content) and might diminish the difference in effective patch size of the treatments. Moreover, pasture soil surface (vegetation cover, surface compaction, micro-topography, soil moisture) is highly heterogeneous and can cause variations in effective patch area. In experimental studies, urine was often applied uniformly on areas larger than the measurement chamber or in chambers inserted into the soil (Da Silva Cardoso et al. 2016; Luo et al. 2019) and thus certainly not covering the effective naturally expanding patch area and changing the emission behaviour. For this reason, a mobile and unframed fast-box chamber of a dimension (80 × 80 cm) larger than the expected effective patch area was used in our study.

### Urine volume had no significant effect on EF_urine_

Except in experiment U2, no effect of the urine application on soil surface WFPS was observed at the day of application (not shown here). Furthermore, there was no significant effect of different amounts of urine (containing different or equal amounts of N) on surface WFPS (not shown here) and on the EF_urine_ values (Table 2). Also, the addition of 2 L water in U4 showed no increase of N_2_O emissions at application day (not shown here), though WFPS level was already high (0.9) before water application. Da Silva Cardoso et al. (2016) observed, in contrast, a significantly decreasing EF_urine_ with increasing urine volume. They suggested a deeper infiltration of urine for higher urine volumes reducing the proportion of applied N in the top-soil layer being used for N_2_O production. However, the results of Da Silva Cardoso et al. (2016) were derived from a single application experiment. Sordi et al. (2014) and van Groeningen et al. (2005b) applied differing urine volumes (having same N concentration and having same total N) at different timings and observed a decreasing EF_urine_ only in single application experiments. Initial soil infiltration of urine is determined by soil properties like soil texture or antecedent soil moisture conditions. As soil moisture and infiltration capacity fluctuates seasonally, the results of singularly carried out application experiments should be regarded with suspicion.

### Urine composition did not affect EF_urine_

To control cattle urine N concentrations, synthetic urine was predominantly used in this study. A variety of artificial urine types have been used in studies including simple urea solutions in water (Petersen et al. 2005) to more complex solutions (Kool et al. 2006b; Bell et al. 2015; Cardenas et al. 2016). Typically, urea forms the largest fraction of N ranging from 52 to 94% of total urine N for diary cattle in temperate regions (Dijkstra et al. 2013). Other nitrogenous compounds include allantoin, hippuric acid, uric acid, creatine and creatinine. In U9, we compared three different synthetically produced urine mixtures containing the same total N amount (i.e., 1) 100% urea-N, 2) 91% urea-N, 9% hippuric acid-N, and 3) 76.7% urea-N, 5.1% hippuric acid-N, 14.1% allantoin-N, 0.8% uric acid-N, 3.2% creatinine-N and found no significant difference in cumulative N_2_O emissions and EF_urine_ values (Table 2). However, synthetic urine composition 1 showed a less intense increase of cumulative N_2_O emissions after urine application (not shown here) and cumulative emissions tended to be smaller than for the other two treatments. This could have been caused by the higher urine pH (Table 1) that is supposed to temporally affect the soil pH and soil mineral N dynamics.

In the three experiments U2, U5 and U9, real and synthetic urine were applied in parallel. A direct comparison of cumulative N_2_O emissions between the treatments was not possible in this study as N concentrations differed. However, assuming that urine volume and N concentration have no effect on the EF_urine_ value (see previous paragraphs), differences of EF_urine_ values can be attributed to differences in the compositions. The observed results were not consistent since U2 showed a higher EF_urine_ value for the real urine while U5 and U9 gave higher values for the synthetic urine.

Kool et al. (2006a) reported that a synthetic urine equal to our standard composition (91% urea-N, 9% hippuric acid-N) was able to simulate real urine with the same N content with regards to its N_2_O emission magnitude. For this reason, they recommended to use synthetic urine mixtures including hippuric acid. However, they also observed deviations in terms of soil NH_4_^+^ and thus it is recommended to use real urine if possible for studying specific soil processes.

### Precipitation and WFPS were main drivers of N_2_O emissions and EF_urine_

Environmental conditions like precipitation and soil WFPS are typically recorded during measurement campaigns and linked to N_2_O emissions (Krol et al. 2016; van Groeningen et al. 2005b). However, contrary to our study these measurements were conducted individually or in a few repetitions. Due to repeated measurement campaigns, covering changing meteorological conditions, we were able to explain the temporal variability of N_2_O emissions and the EF_urine_ by WFPS and precipitation.

WFPS is a proxy for the aeration status of the soil controlling microbial N_2_O producing processes. Classically, the relation between WFPS and N_2_O production is described with an optimum curve reaching maximum values of N_2_O production at 0.6–0.8 WFPS (Congreves et al. 2019; Davidson et al. 1991; Butterbach-Bahl et al. 2013). However, N_2_O production after urine application is elevated for a longer period, e.g. 30–50 days in Pal Singh et al. (2021) and 10–60 days in Krol et al. (2016) comparable with our results. In our study, averaged WFPS over a period of 30 days showed the best fit with derived EF_urine_ values. The relation was linear, indicating that EF_urine_ values increase proportionally with averaged WFPS. Certainly, the averaged value does not account for variations of soil moisture within averaging time, and so called “hot moments” (Barrat et al. 2020). With rapid changes in soil moisture, N_2_O emissions can increase abruptly. Barrat et al. (2020) found larger hot moments for larger differences between the dry and wet states of the soil. In addition, the hot moment was larger, the larger the WFPS after rewetting. In our study, changes in WFPS were more distinct in summer than in spring or autumn and can be explained by more excessive precipitation events and higher evapotranspiration in the warmer summer. However, N_2_O emissions in hot moments might be balanced by drier periods when activity of N_2_O producing microbes is inhibited.

Precipitation is known to regulate soil moisture and studies usually link higher precipitation in wet seasons to more N_2_O production (Chadwick et al. 2018; Zhu et al. 2021; Krol et al. 2016). Our results indicate an optimum of the EF_urine_ value at cumulative precipitation of 83 mm. Precipitation amounts exceeding the optimum may lead to higher NO_3_- leaching and higher rates of complete denitrification, thereby reducing N_2_O production in the top soil. López-Aizpún et al. (2020) observed a decreasing EF_urine_ with increasing air temperature, explained by evaporation being positively correlated with soil temperature and thus leading to lower soil moisture. However, we did not see an effect of soil temperature on EF_urine_.

### Temporal variability of EF_urine_ was not linked to seasons

EF_urine_ values were derived from 10 experiments with different timing of urine application during the grazing season covering differing conditions of precipitation, soil WFPS and temperature, and ranged from 0.17 to 2.05%. In consideration of the temporal variability of EF_urine_ values, a disaggregation by season has been considered in some publications. In temperate climates, Chadwick et al. (2018) and Anger et al. (2003) measured higher emissions in spring, whereas van Groeningen et al. (2005b), Maire et al. (2020) and Krol et al. (2016) saw higher emissions in autumn exhibiting an inconsistent seasonal effect. Most of the studies conducted only one experiment per season, whereas in our study at least two repetitions per season were carried out showing a wide range in event related EF_urine_ values within the season. Furthermore, the simulation of EF_urine_ values via derived precipitation model demonstrated no seasonal pattern of cumulative precipitation and corresponding EF_urine_ values (Fig. 4). Our results demonstrate that a seasonal disaggregation is not appropriate and rather environmental conditions on a smaller timescale (20–30 days) determine the EF_urine_.

### Mean observed EF_urine_ was close to the IPCC default value

The simulated average EF_urine_ over the last two decades (Fig. 4) was 0.67%, similiar to the average EF_urine_ of the experimental data (0.73%). The IPCC suggests a similar value of 0.77% for urine patches in wet climates (IPCC 2019). Assuming an EF_dung_ two to three times lower than the EF_urine_ (Cai and Akiyama 2016; Voglmeier et al. 2019; Chadwick et al. 2018), the resulting EF_3_ for all pasture excreta will be around 0.50% for our study. This value is close to the new IPCC default EF_3_ of 0.4% and to the disaggregated EF_3_ of 0.6% for wet climates, but much lower than the old IPCC default EF_3_ of 2%.

For central Europe and Switzerland in particular, the data basis of measured EF_3_ is very sparse. Voglmeier et al. (2019) also conducted measurements of N_2_O from urine and dung patches on a dairy pasture in Western Switzerland. Their derived EF_3_ (0.79%) and EF_urine_ (1.13%) were higher than in our study. However, in contrast to our study, Voglmeier et al. (2019) derived EF values from only a four month period and the soil texture was different potentially affecting the formation of N_2_O. The effects of soil properties like soil texture, pH or C/N ratio on N_2_O emissions from urine patches have been studied only marginally (Zhu et al. 2020; Rochette et al. 2014) up to now.

## Conclusion

In the present study EF_urine_ values were not significantly affected by the urine N input, patch area, volume and composition. This supports the comparability and averaging of EF_urine_ results of different experimental studies using differing patch characteristics. However, our study did not account for soil properties and climate potentially influencing urine N cycling and the effect of urine patch characteristics. To quantify EF_urine_ and cumulative urine emissions under other site conditions, we recommend to parametrize the second-order polynomial precipitation model and the linear WFPS model locally to adapt to site conditions.

The results of this study corroborate the use of the IPCC default value of 0.77% for the urine N input by grazing cattle in Switzerland. However, our results were obtained only from one site. To better justify the use of the IPCC default EF_urine_ value or to implement a country-specific EF_urine_ in Switzerland, more measurements at different locations or the use of a suitable process-based model to simulate the effect of different soil conditions in combination with other factors are necessary. Future studies also need to focus on dung patches to test whether the EF_dung_ falls into the range of the IPCC default value.
